# Association between obesity and depression in patients with diabetes mellitus type 2; a study protocol

**DOI:** 10.12688/f1000research.5995.1

**Published:** 2015-01-09

**Authors:** Eduardo De la Cruz-Cano, Carlos Alfonso Tovilla-Zarate, Emilio Reyes-Ramos, Thelma Beatriz Gonzalez-Castro, Isela Juarez-Castro, Maria Lilia López-Narváez, Ana Fresan

**Affiliations:** 1 División Multidisciplinaria de Comalcalco, Universidad Juárez Autónoma de Tabasco, Comalcalco, Tabasco, 86650, Mexico; 2 División Académica Multidisciplinaria de Jalpa de Méndez, Universidad Juárez Autónoma de Tabasco, Cunduacán, Tabasco, 86690, Mexico; 3 División Académica de Ciencias de la Salud, Universidad Juárez Autónoma de Tabasco, Villahermosa, Tabasco, 86100, Mexico; 4 Secretaría de Salud, Hospital General de Yajalón, Yajalón, Chiapas, 29930, Mexico; 5 Departamento de Investigaciones Clínicas, Instituto Nacional de Psiquiatría Ramón de la Fuente Muñiz, Colonia San Lorenzo Huipulco, Delegación Tlalpan, 14370, Mexico

**Keywords:** Diabetes Mellitus type 2; obesity; body mass index; depression.

## Abstract

**Background**: Diabetes mellitus and depression are highly prevalent conditions throughout the world and have significant impact on health outcomes. It has been estimated that diabetes mellitus type 2 affects about 246 million people in the world; nevertheless, incidence varies among countries. There is evidence that depression is associated with a poor metabolic control in patients with type 2 diabetes mellitus that present other health problems (such as hypertension and obesity). The aim of this study protocol is to determine if obesity increases the risk for depression in patient with diabetes type 2.

**Methods**: The analysis will be reported following the Preferred Reporting Items for Systematic Reviews and Meta-Analyses (PRISMA).The studies suitable for inclusion will be assessed by the Newcastle-Ottawa Scale (NOS) to determine their methodological quality. To identify the studies of interest, we will search on PubMed and EBSCO databases. We will use the following keyword combinations: "Diabetes Mellitus type 2 AND obesity AND depression", "depression AND Diabetes Mellitus type 2", "Diabetes Mellitus type 2 AND body mass index cross sectional study", "depression AND obesity cross-sectional study". Causes for exclusion will be publications that studied patients diagnosed with diabetes mellitus type 1; articles that focused on the treatment and complications of diabetes mellitus type 2; publications that have studied other clinical or psychiatric conditions (for instance, seizure disorder or history of schizophrenia, bipolar disorder, psychotic symptoms or dementia).

**Conclusion: **The results of this study will form the basis for a better understanding of the association between obesity and depression in patients with diabetes mellitus type 2, and will allow development of prediction tools and better interventions. It is evident that several modifiable and non-modifiable risk factors play an important role in the pathogenesis of diabetes among population. Currently, evidence for the deleterious effects of diabetes mellitus type 2 are based on cross-sectional or other observational designs. Therefore, this study will have important implications for future research and public health guidance.

## Background

Diabetes and depression are highly prevalent conditions throughout the world and have significant impact on health outcomes. Diabetes mellitus is a chronic-degenerative disease, characterized by high levels of blood glucose ^1– 3^. It has been estimated that diabetes mellitus type 2 affects about 246 million people in the world ^4^; nevertheless, incidence varies among countries ^5, 6^. The International Diabetes Federation has anticipated an increase of 366 million people by 2030, giving a total of 552 million people with diabetes type 2 in the world ^6, 7^.

The diabetes type 2 is a complex disease, where hereditary and metabolic factors interfere ^8, 9^. Literature suggests there is a correlation between type 2 diabetes and mood alterations such as depression and neuropsychiatric disorders; for instance, major depressive disorder ^10, 11^, schizophrenia ^12, 13^, mild cognitive impairment ^14, 15^ and suicidal behavior ^16^. It also has been observed that depression could cause an increase in all-cause mortality risk (approximately 70%) ^17^; it is also the most common mental disorder and generates a great impact on people and society in terms of suffering, disability and economic costs, a phenomenon that seems to occur in many parts of the world; in this context, it has been reported that depression affects 350 million people worldwide ^18^; for example, a research by Talbot *et al.* suggests that depression is not only a direct consequence of diabetes; depression may also be a risk factor for the onset of diabetes type 2 ^19^. Patients with diabetes mellitus type 2, often present a careless attitude towards their disease, resulting in metabolic decompensation, with high and low glycemic levels which could trigger mood alterations ^20, 21^. Diabetes mellitus 2 is also associated with a higher risk of comorbid depression compared with the general population ^22^. It has been suggested that diabetes type 2 could be conditioned by depression, anxiety or anguish ^23– 25^; nevertheless, the reason for this association is not clear ^26, 27^. The neurobiological mechanisms that could explain the association between depression and diabetes mellitus type 2 ^28^ could include 1) the alterations involved in the metabolism of biogenic amines (serotonin and norepinephrine), from the adrenal-pituitary-hypothalamus axis (by increasing cortisol) ^28– 30^; 2) trophic agents such as Brain Derived Neurotrophic Factor (BDNF) through Glycogen Synthase Kinase-3 (GSK-3) ^31, 32^. The GSK-3 is a serine/threonine protein kinase that mediates the addition of phosphate molecules into serine and threonine amino acid residues. It consists of two isoforms, α and β ^33, 34^. It is possible that an over activation of GSK-3 play an important role in the pathogenesis of the development of schizophrenia and mood disorders such as bipolar disorder and major depression in patients with diabetes mellitus type 2 ^35, 36^. Furthermore, it has been suggested that the presence of metabolic alterations in patients with diabetes type 2 such as obesity, could increase the severity of depression ^37– 40^. The distinct mechanisms that link obesity to insulin resistance and diabetes mellitus type 2 are related to an increased production of adipokines and more adipose tissue as a result ^41, 42^; these molecules are involved in many clinical manifestations of diabetes mellitus type 2 and they are also associated with arterial hypertension and cardiovascular disease ^43^. First, the adipose tissue of the obese patient becomes resistant to the action of insulin due to the effect of some of these adipokines; for instance, the tumoral necrosis factor alpha (TNF-α) and interleukine-6 (IL-6) ^44^. Secondly, this resistance occurs in other tissues; therefore, insulin and glucose levels increase. This increase, along with high adipokines levels (that occur in diabetes mellitus type 2), lead to different adverse events, such as endothelial dysfunction ^45^, increase in oxidative stress ^46^, impairments in lipoprotein metabolism and increase in blood pressure ^47^. For a review see Antuna puente *et al.* ^48^. For example, a research by Svenningsson *et al.* suggests an association between depression and obesity in patients with diabetes mellitus type 2 in both genders; this study reported that at least one in five men and one in three women showed depression in diabetic type 2 patients with obesity ^49^. Recently, a report showed that there is a positive association between having a high body mass index and the risk to develop diabetes mellitus type 2 ^50^. In general, literature shows evidence that depression is associated with metabolic disorders in patients with type 2 diabetes mellitus ^27^.

In this work we will focus on searching a correlation between obesity and depression in patient with diabetes type 2. The aim of this study is to determine if obesity in patients with diabetes type 2 increases the risk of depression.

**PICOT QUESTION**: Does obesity increase the risk of depression in Diabetes Mellitus type 2 patients?

## Methods/Design

The study protocol will be conducted and reported in compliance with the Preferred Reporting Items for Systematic Reviews and Meta-Analyses (PRISMA) guidelines ^51^. In accordance with the guidelines, our study protocol was registered with the International Prospective Register of Systematic Reviews (PROSPERO) ^52^, on 08 October, 2014 (registration number CRD42014014034).

## Literature search strategy

**Figure 1.**
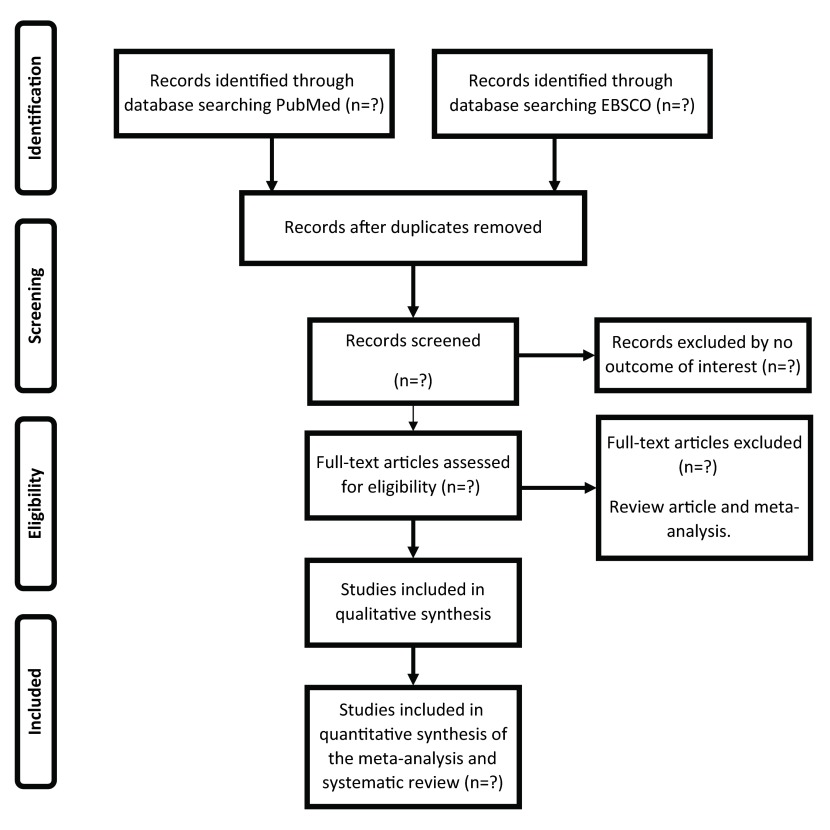
Flow chart for the search strategy and the inclusion/exclusion criteria used in the meta-analysis and systematic review.

The selection of publications and the reporting of results for the study protocol will be conducted in accordance with the PRISMA guidelines ^51^. We will search on PubMed and EBSCO databases. We will further scan reference lists in relevant reviews and publications retrieved for the purpose of our study protocol. There will be no initial limit on the date of publication. We will use the following keyword combinations: "Diabetes Mellitus type 2 AND obesity AND depression", "depression AND Diabetes Mellitus type 2", "Diabetes Mellitus type 2 AND body mass index AND cross sectional study", "depression AND obesity AND cross-sectional study". The bibliography of the articles chosen will also be examined in order to find more articles that might not be on the electronic databases. We will only include case-control, cross-sectional and cohort studies. The planned procedure is illustrated in Figure 1.

## Eligibility criteria

Titles and abstracts will be screened for eligibility according to the following inclusion and exclusion criteria.

### Inclusion criteria

For the purpose of this study protocol will be included publications in English language that examined the relation of body mass index (BMI > 30 kg/m ^2^) and severity of depression in patients with diabetes type 2.

### Exclusion criteria

Causes for exclusion will be: publications that studied patients diagnosed with diabetes mellitus type 1; articles that focused on treatment and/or complications of diabetes mellitus type 2; publications or clinical trial that have focused on treatment of metabolic and psychiatric disease (for instance, mood stabilizers, neuroleptic, antidepressant, benzodiazepines, seizure disorder, history of schizophrenia, bipolar disorder, psychotic symptoms and dementia).

## Type of studies

This study protocol will include case-control, cross-sectional and existing cohort studies up to date.

## Type of participants

The participants will be adults (aged 18 years and over), diagnosed with diabetes mellitus type 2. For the purpose of this review, only overweight and obese type 2 diabetes mellitus patients with symptoms of depression will be included.

## Screening

First, Two independent reviewers will read the titles of all the citations retrieved from the electronic database searches and removed all citations that are clearly not related to our study. Next, the abstract will be assessed to determine if the study satisfies the inclusion criteria. If from the abstract it is unclear whether the selection criteria are met or not, the full article will be scanned. Any discrepancy for inclusion will be discussed with a third or fourth author. Once the appropriate articles have been chosen for further analysis, two or three authors (independently) will be involved in the assessment of each article and data extraction. Further studies may be excluded as a result of not being relevant for our study. Further studies may be included through searching the reference lists in publications selected for the review. All the studies included will be read in detail and the relevant information extracted. The degree of agreement between the observers will be calculated by the Kappa coefficient; the studies that cause disagreement will be reviewed again, then the observers will decide the inclusion/exclusion together. The studies selected will be evaluated for quality and incorporation of gender perspective. Studies deemed for inclusion will be scored for methodological quality using the Newcastle-Ottawa Assessment Scale (NOS) ^53^. Results will be analyzed using a narrative synthesis. To give more support to our analysis, we will consider the GRADES scale procedures ( http://www.gradeworkinggroup.org).

## Analysis of results

A descriptive synthesis of important characteristics will be undertaken independently, including, author, year study, sample characteristics, type of study design, length of follow-up (for cohort studies), exposure variable characteristics, dependent variable characteristics, method used to ascertain diabetes status and body mass index; assessment of depression, relative risk or equivalent associated with diabetes mellitus type 2 and obesity. A quantitative synthesis of effects will not be attempted because of substantial methodological heterogeneity among studies.

Whenever possible, adjusted relative risk (RR) or equivalent and associated 95% CI will be extracted directly from studies. For studies that present RR by subgroups (for example, relative risk associated with Body Mass Index, kg/m ^2^ ≥30) the data for each subgroup will be additionally extracted. Authors will be contacted via email for any missing relevant information. Also, data will be analyzed descriptively. The systematic review will be presented in tables comparing quality measurements and the data previously mentioned.

## Discussion

The aim of this study protocol is to verify if there is a direct relation between depression and obesity in patients diagnosed with Diabetes Mellitus type 2, with the aim of improving the treatment of these patients, through an updated and quantitative estimate of the risk of depression associated with obesity. This study protocol will include a wide number of study designs; therefore, a subgroup analysis will be performed, to understand the relation between depression and obesity in patients with type 2 diabetes according to type of study. Furthermore, literature suggests that age is associated with depression, as well as other emotional alterations ^54, 55^; therefore, age could also influence patients with obesity to develop depression. Nevertheless; up to today, there are no-systematic reviews that search for this association. It is important to know if there is a connection between a high body mass index (BMI) and emotional alterations of diabetes mellitus type 2 patients ^56, 57^. Finally, depression and obesity appear to be linked with poorer behavioral management of diabetes and glycemic control; therefore, the need for comprehensive interventions worldwide that target depression in conjunction with the type 2 diabetes mellitus management. The findings from this study protocol will be widely disseminated through discussions with stake-holders, publication in a peer-reviewed journal and a conference presentation. This study protocol on diabetes and depression will bring to light knowledge gaps in the area and will offer directions for future researches.

## Abbreviations

BDNF: Brain Derived Neurotrophic Factor; BMI: Body Mass Index; CRH: Corticotropin-Releasing Hormone; GSK-3: Glycogen Synthase Kinase-3; IL-6: Interleukine-6; NOS: Newcastle-Ottawa Scale; PRISMA: preferred reporting items for systematic reviews and meta-analyses; PROSPERO: Prospective Register of Systematic Reviews; RR: risk ratio; TNF-α: Tumoral Necrosis Factor alpha; T2DM: Type 2 Diabetes Mellitus.
