# Supplementary material for: Association between obesity and depression in patients with diabetes mellitus type 2; a study protocol
Source: F1000Res. 2015 Jan 9;4:7. [Version 1] doi: 10.12688/f1000research.5995.1 (PMC4358412; doi:10.12688/f1000research.5995.1)
Supplement: Peer review history

# Peer review: Referee response for version 1

**DOI:** 10.5256/f1000research.6413.r7781

**Published:** 2015-03-13

**Authors:** Janusz Rybakowski

**Affiliations:** 1 Department of Adult Psychiatry, Poznan University of Medical Sciences, Poznan, Poland

The aim of this paper is to make a systematic review and meta-analysis of the association between obesity and depression in patients with diabetes mellitus type 2. The results of the study may allow development of prediction tools and better interventions in such condition. The rationale for the study is provided and the methods proposed seem correct.

My only reservation is that, in psychiatric patients and in general population, obesity has been found to be mostly associated with a specific kind of depression, i.e. depression with atypical features or atypical depression ( Levitan *et al*, 2012, Glaus *et al*, 2013, Chou and Yu, 2013). Therefore, I would suggest that the authors may take this kind of depression into account in their analysis.

I have read this submission. I believe that I have an appropriate level of expertise to confirm that it is of an acceptable scientific standard.

# Peer review: Referee response for version 1

**DOI:** 10.5256/f1000research.6413.r7266

**Published:** 2015-01-16

**Authors:** Hans-Peter Volz

**Affiliations:** 1 School for Psychiatry and Psychotherapy, Hospital for Psychiatry, Psychotherapy and Psychosomatic Medicine Schloss Werneck, Bavaria, Germany

In the study plan of De la Cruz-Cano and co-workers the parameters for a structured literature research about the relationship between diabetes type II, obesity and diabetes are reported. The rational for this planned investigation is stated very clearly, the methods proposed seem adequate, the purpose of the planned investigation is highly welcomed.

Therefore the study protocol should be indexedin order to stimulate the discussion about this very important topic and the planned literature review.

I have read this submission. I believe that I have an appropriate level of expertise to confirm that it is of an acceptable scientific standard.
